# Senolytic treatment with dasatinib and quercetin does not improve overall influenza responses in aged mice

**DOI:** 10.3389/fragi.2023.1212750

**Published:** 2023-06-16

**Authors:** Blake L. Torrance, Andreia N. Cadar, Dominique E. Martin, Hunter A. Panier, Erica C. Lorenzo, Jenna M. Bartley, Ming Xu, Laura Haynes

**Affiliations:** ^1^ University of Connecticut Center on Aging, Farmington, CT, United States; ^2^ Department of Immunology, Farmington, CT, United States; ^3^ Department of Medicine, Farmington, CT, United States; ^4^ Department of Genetics and Genome Sciences, Farmington, CT, United States

**Keywords:** T-cells, cellular senescence, senolytics, influenza, T-cell memory

## Abstract

Age is the greatest risk factor for adverse outcomes following influenza (flu) infection. The increased burden of senescent cells with age has been identified as a root cause in many diseases of aging and targeting these cells with drugs termed senolytics has shown promise in alleviating many age-related declines across organ systems. However, there is little known whether targeting these cells will improve age-related deficits in the immune system. Here, we utilized a well characterized senolytic treatment with a combination of dasatinib and quercetin (D + Q) to clear aged (18–20 months) mice of senescent cells prior to a flu infection. We comprehensively profiled immune responses during the primary infection as well as development of immune memory and protection following pathogen reencounter. Senolytic treatment did not improve any aspects of the immune response that were assayed for including: weight loss, viral load, CD8 T-cell infiltration, antibody production, memory T cell development, or recall ability. These results indicate that D + Q may not be an appropriate senolytic to improve aged immune responses to flu infection.

## Introduction

It is well established that older adults, those over the age of 65, bear the greatest burden of mortality following influenza (flu) infection, comprising 83% of deaths during the 2021–22 season (CDC, 2022). Similarly, older adults are at greatest risk for adverse outcomes in the context of other respiratory viral infections like COVID-19, where over 75% of deaths have occurred among older adults (CDC, 2023). The reasons underpinning this vulnerability are manifold, but much of this disparity is related to the systemic age-related declines in the makeup and function of the immune system and its response to pathogens.

Aging results in alterations in the functionality of both the innate and adaptive arms of the immune system (Linton and Dorshkind, 2004; Shaw et al., 2013). Within the adaptive immune system, CD8 T-cells are the primary mediators of viral clearance via the direct cytotoxic killing of infected cells. With age, CD8 T-cells cytotoxic activity declines and, therefore, the ability to efficiently clear virus also declines (Jiang et al., 2009; Quinn et al., 2018). Similarly, the differentiation of both CD4 and, although to a lesser extent, CD8 T-cells from effector to memory subtypes is also suboptimal with age and the capability of these cells to respond to subsequent pathogen encounter is deleteriously affected (Haynes et al., 2003; Nikolich-Žugich, 2018). This, combined with known age-associated deficits in mechanisms of T-cell activation, proliferation, and function, leaves older adults at great risk for adverse outcomes following flu infection. Studies utilizing mouse models have demonstrated that aged mice have delayed viral clearance, delayed and diminished CD8 expansion, and prolonged inflammation in response to flu infection (Lefebvre et al., 2016). It is of great importance to investigate potential therapeutics that could alleviate these age-related deficits and improve immune responses among older adults and enhance their protection against respiratory viruses.

Recently, a role for cellular senescence and the accumulation of senescent cells has emerged as underlying cause of a great number of age-related declines in nearly all organ systems. Cellular senescence is a mostly irreversible state of proliferative arrest enforced by expression of cyclin-dependent kinase inhibitors like p21^Cip1^ and p16^INK4A^ (Gasek et al., 2021). Induction of the senescent cell fate occurs following an insult or stressor. Importantly, these cells can become resistant to apoptosis upon activation of various senescent cell associated anti-apoptotic pathways via signaling through, among other pathways, PI3K or BCL-2 family members (Kirkland and Tchkonia, 2017). Senescent cells also remain metabolically active and secrete a heterogenous mixture of generally proinflammatory cytokines including IL-6, TNF-α, IL-1β, CCL2, as well as some profibrotic factors like TGF-β as a part of the senescence associated secretory phenotype (SASP) (Coppé et al., 2008).

Approaches using drugs to target senescent cells, termed senolytics, in order to alleviate the effects of aging have been fruitful in many contexts. The first and most well described senolytic treatment is a combination therapy consisting of dasatinib (D), an FDA-approved tyrosine kinase inhibitor, and quercetin (Q), a natural plant derivative (Zhu et al., 2015). This combination is well suited for our studies due to its demonstrated efficacy in clearing senescent cells in the lungs of aged mice (Schafer et al., 2017). This treatment has been shown to improve many chronic diseases of aging including obesity (Palmer et al., 2019), cardiovascular disease (Roos et al., 2016; Lewis-McDougall et al., 2019), renal dysfunction (Kim et al., 2021) and many others (Chaib et al., 2022). D + Q treatment was also shown to improve overall longevity and healthspan (Xu et al., 2018). Importantly, D + Q is the only senolytic treatment that has been demonstrated to be safe and effective in human trials with older adults to date (Hickson et al., 2019; Justice et al., 2019). Aside from D + Q there are other senolytic treatments that have been proven effective in mouse models including navitoclax (Zhu et al., 2016) and fisetin (Yousefzadeh et al., 2018). Despite the great promise demonstrated by many studies utilizing senolytics, their effects on the immune system remain unclear.

Our group was among the first to describe the effects of senolytics on aged immune responses using D + Q (Lorenzo et al., 2022). We found that senolytic treatment induced favorable alterations in CD4 helper subset differentiation patterns in a cell extrinsic manner, likely related to SASP factors. Another study, using fisetin, described an improvement in survival following coronavirus infection among aged mice receiving the senolytic (Camell et al., 2021). Notably, these studies are limited in scope to the response to a primary infection. Especially in the context of seasonal flu infection in older adults, studies evaluating the potential efficacy of senolytic to improve their immune responses must consider secondary responses and the development of protective immune memory. The overall benefits of senolytic treatment in improving antiviral responses with age is still understudied and has been largely ignored despite growing interest in utilizing senolytics in the clinic. Here, we sought to test the efficacy of D + Q treatment on the overall response to flu infection in aged mice.

## Materials and methods

### Mice

All experiments utilized aged (18–20 months) C57BL6/J male mice generously provided by the National Institute on Aging Rodent Colony and housed at UConn Health. All mice underwent examination at the time of sacrifice and animals with gross pathology (e.g., visible tumors) were excluded from analysis. All mice were housed in a climate-controlled environment and were provided standard chow and water *ad libitum*. All mice were cared for in accordance with the recommendations in the Guide for the Care and Use of Laboratory Animals of the National Institutes of Health. All procedures were approved by the UConn Health Institutional Animal Care and Use Committee (IACUC).

### Senolytic treatment

**FIGURE 1:**
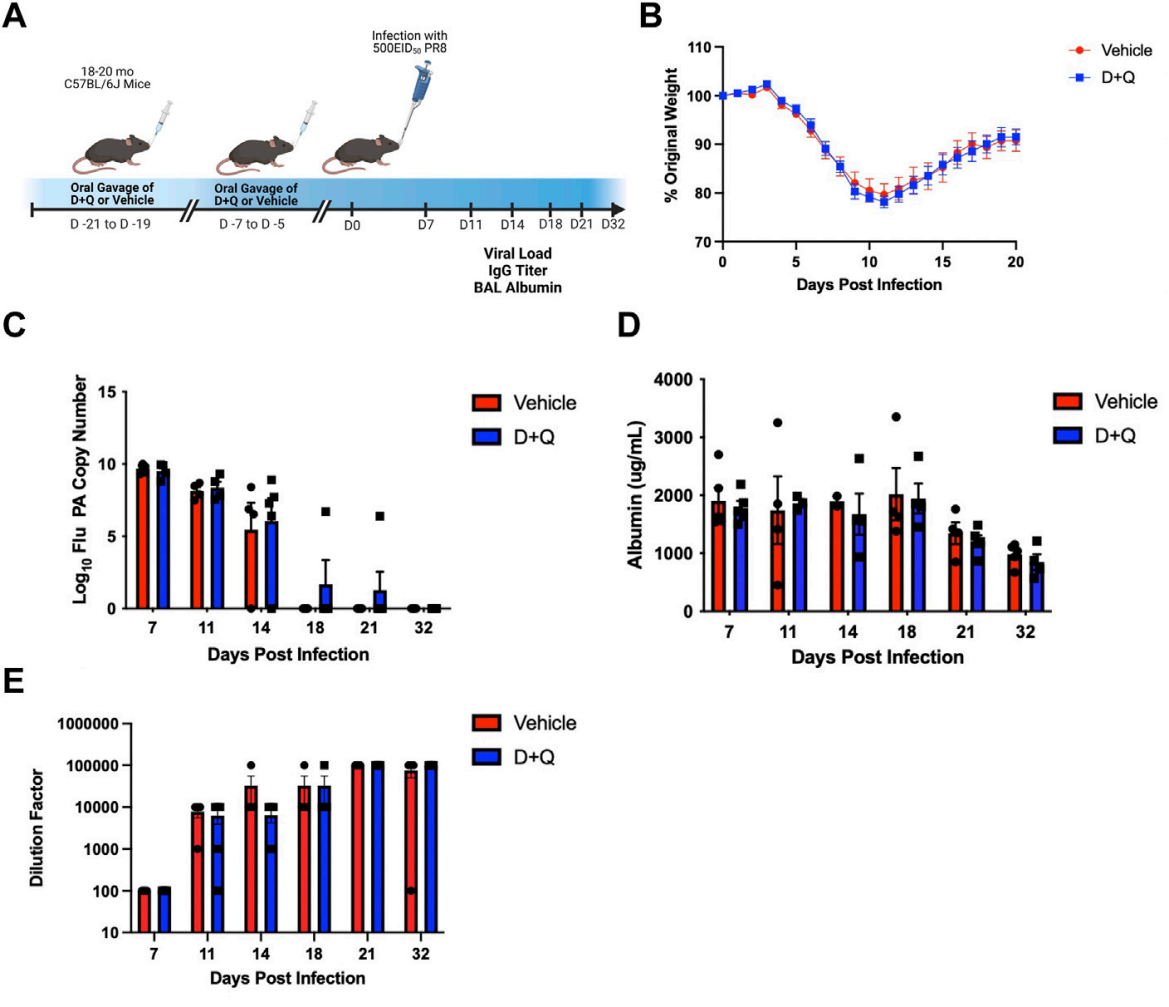
D + Q Does Not Improve Gross Metrics of Disease Progression Following Primary Flu Infection. 18–20 months male wild type C57BL/6J (B6) mice were treated intermittently with senolytic drug combination dasatinib plus quercetin (D + Q) or vehicle control. Following a rest period of 5 days, mice were infected with a sublethal dose of PR8 H1N1 flu **(A)**. Percent of body weight lost throughout the infection was monitored **(B)**. Viral replication was quantified by RT-qPCR for flu acid polymerase (PA) gene copies **(C)**. Albumin levels in the bronchoalveolar lavage (BAL) were measured as a metric of infection-induced damage **(D)**. Levels of systemic whole PR8-specifc IgG was quantified from serum **(E)**. Data are presented as mean ± standard error of the mean (SEM). Two-way ANOVA was performed using Šidák’s test for multiple comparisons. Results were considered significant at *p* < 0.05. Comparisons between treatment groups are nonsignificant unless otherwise indicated. *N* = 4–5 per group per timepoint.

Mice were treated via oral gavage with 5 mg/kg/day dasatinib (D) and 50 mg/kg/day quercetin (Q) or an equal volume of vehicle control consisting of 10% ethanol, 30% polyethlene glycol, and 60% Phosal 50PG. As illustrated in Figure 1A, mice were treated for three consecutive days, allowed to rest for 1 week, then treated again for three consecutive days before resting for 5 days prior to flu infection. The half-lives for D and Q are 4 and 11 h, respectively (Graefe et al., 2001; Christopher et al., 2008). Thus, our rest period ensures complete clearance of both drugs prior to infection. This intermittent dosing strategy has been frequently used when administering D + Q and has been shown to increase lifespan and ameliorate age-related dysfunction (Xu et al., 2018).

### Viral infection

Mice were anesthetized using isoflurane and intranasally infected with a sublethal dose of either H1N1 influenza virus A/Puerto Rico/8/34 (PR8) or H3N2 influenza virus A/HKx31 (x31). Doses for PR8 were either 500 EID_50_ in 50uL PBS for primary infection experiments or 700 EID_50_ in 70uL of PBS for rechallenge studies. For memory experiments utilizing x31, mice were given 3000 EID_50_ in 50uL PBS. Mice were monitored regularly to assess percent weight lost as an indication of infection progress. Moribund mice and those which lost more than 30% of original body weight were euthanized.

### Viral load

Following sacrifice, lungs were immediately flash frozen in liquid nitrogen. Lung tissue was homogenized using a handheld homogenizer (Pro Scientific) and RNA was isolated via standard trizol/chloroform (Invitrogen Life Technologies and Sigma Aldrich, respectively) extraction per the manufacturer’s protocol. cDNA was synthesized using iScript cDNA synthesis kit (Bio-Rad) using the manufacturers protocol. Viral load was determined by RT-qPCR for PR8 acid polymerase (PA) gene compared to a standard curve of known PA copy numbers as previously published (Jelley-Gibbs et al., 2007; Keilich et al., 2020). The following primer and probe were used: forward primer, 5′-CGG​TCC​AAA​TTC​CTG​CTG​A-3′; reverse primer, 5′- CAT​TGG​GTT​CCT​TCC​ATC​CA-3′; probe, 5′-6-FAM-CCAAGTCATGAAGGAGAGGGAATACCGCT-3′ (Integrated DNA Technologies).

### Antibody quantification

Serum was obtained from whole blood collected via cardiac puncture upon sacrifice. Samples were serially diluted 10-fold. Diluted serum was transferred to microplates coated with whole PR8 viral particles. Using a horseradish peroxidase conjugated to an anti-IgG antibody (Southern Biotech), levels of flu-specific IgG were measured via ELISA. Titer was determined at highest dilution which had a measured absorbance at 490 nm over the mean plus standard deviation of blank wells.

### Albumin and cytokine quantification

Bronchoalveolar lavage (BAL) was collected by flushing lungs with 1mL of PBS at time of sacrifice. Samples underwent centrifugation to exclude debris. Concentration of albumin in BAL was determined using an albumin ELISA kit (Abcam) following the manufacturer’s protocol. Cytokine analysis was performed using a magnet-based multiplex ELISA (Millipore) and analyzed using a MAGPIX plate reader (Luminex).

### Tissue processing and flow cytometry

Following sacrifice, lungs were initially mechanically digested. They were then enzymatically (100 U/mL collagenase, Gibco) digested in RPMI media containing 5% fetal bovine serum for 40 min at 37°C. Red blood cells were lysed using ACK lysis buffer (Gibco). For flow cytometry, cells were incubated with Fc block (anti-CD16/32, ThermoFisher) followed by staining with NP_366-374_ H-2D^b^ MHC Class I tetramer (generated by the NIH Tetramer Core Facility). Cells were subsequently stained with surface antibodies and a live/dead fixable violet stain (ThermoFisher). Samples only undergoing surface staining were fixed using 1% paraformaldehyde. Samples undergoing intracellular Granzyme B staining were fixed and permeabilized using a FoxP3/Transcription Factor staining kit (ThermoFisher) prior to incubation with intracellular stain. The following antibodies were used: CD8-PerCP-Cy5.5, CD4-BV711, CD8-APC-Cy7, CD69-FITC, CD103-PerCP-Cy5.5, and Granzyme B-FITC. A Becton Dickinson (BD) LSR II cytometer was used and analysis was performed using FlowJo (BD).

### Statistics

All data are presented as mean ± standard error of the mean (SEM). For time courses shown in Figure 1 and Supplementary Figure S1, a two-way ANOVA using Šidák’s test for multiple comparisons was utilized. For all other comparisons, a Student’s *t*-test was used. Analyses were performed using Prism 8 software (GraphPad). *p*-values <0.05 were considered significant. Comparisons between groups were nonsignificant unless otherwise indicated.

## Results

### Effects of senolytic treatment on primary antiviral response

Since our prior work has shown significant differences in the response to influenza infection in young and aged mice, including slower viral clearance, more dramatic weight loss and increased albumin in the bronchoalveolar lavage (BAL) (Lefebvre et al., 2016), we sought to determine if treating aged mice with senolytics could improve these measures. We treated aged (18–20 months) mice with D + Q to clear senescent cells before a sublethal flu infection (Figure 1A). To assess infection severity, weight loss was measured throughout the infection. As we have previously reported (Bartley et al., 2016), during influenza infection aged mice failed to recover weight to return to baseline (Figure 1B). Importantly, senolytic treatment failed to have any impact on weight loss or recovery. Viral load was similarly unaffected by treatment with senolytics, with both control and treated groups showing similar levels of viral burden throughout the infection (Figure 1C). To assess lung damage following viral infection, albumin levels in the BAL were assessed and we found that senolytic treatment was unable to significantly improve lung pathology and/or the return to homeostasis in this context (Figure 1D). Systemic levels of flu-specific IgG were also unaffected by senolytic treatment (Figure 1E). Levels of cytokines that are known SASP factors (i.e., IL-6, TNF-α, and CCL2) were quantified in the BAL and were not altered by D + Q treatment (Supplementary Figure S1).

**FIGURE 2:**
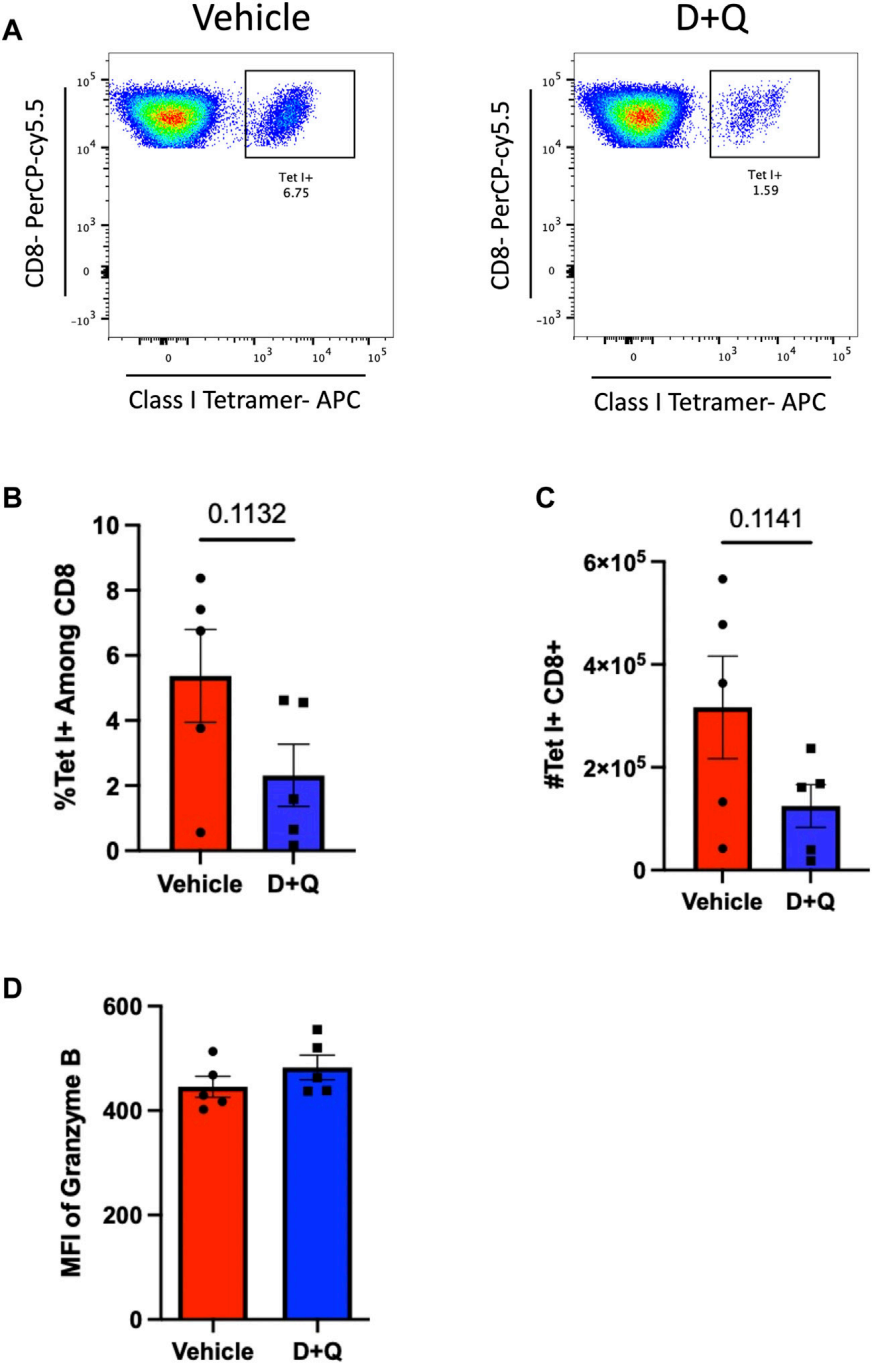
D + Q treatment transiently decreases infiltration of flu-specific CD8 T-Cells following primary infection. 18–20 months male mice were treated with D + Q or vehicle and infected as in Figure 1. Lungs were assayed for infiltrating flu NP-specific CD8 T-cells via flow cytometry **(A)**. Frequency of NP-specific CD8 T-cells was measured at 10 days post infection **(B)**. Numbers of NP-specific CD8 T-cells were also quantified **(C)**. Expression of intracellular Granzyme B was assessed via flow cytometry **(D)**. Data are presented as mean ± standard error of the mean (SEM) and each symbol represents a single animal. Student’s *t*-test was utilized for all experiments, with results being considered significant at *p* < 0.05. Comparisons are nonsignificant unless otherwise indicated. *N* = 5 per group.

Our previous work showed that D + Q treatment significantly impacted the differentiation of CD4 helper T-cell subsets, reducing frequency of FoxP3-expressing regulatory T-cells (Tregs) (Lorenzo et al., 2022). While these changes were observed in the subsets of flu-specific CD4 T-cells, the frequency of flu-specific CD4 T-cells were unaffected. Here, when we examined the CD8 T-cell compartment (Figure 2A), we found that D + Q treatment induced a nonsignificant (*p* = 0.1132) reduction in the frequency of flu nucleoprotein (NP)-specific CD8 T-cells in the lungs at 10 days post infection (DPI) (Figure 2B). Further quantification of numbers of infiltrating NP-specific CD8 T-cells revealed a similar nonsignificant decrease in D + Q treated groups (*p* = 0.1141) (Figure 2C). This nonsignificant reduction is transient as no trends were observed at 12 DPI in our prior studies. Therefore, D + Q was unable to improve the established decline of expansion in flu-specific CD8 T cells with aging at this time point (Po et al., 2002). Additionally, no differences were seen in phenotype as memory precursor or short-lived effector subsets defined via CD127 and KLRG-1 expression (Lorenzo et al., 2022). In order to more fully assess the functionality of these cells, Granzyme B expression was quantified (Figure 2D). Similarly, no differences were detected between groups. It is possible that administration of D + Q has opposing effects in T-cells when comparing the CD4 and CD8 compartments, where D + Q can improve CD4 subset differentiation while not affecting the expansion of flu NP-specific CD8 T-cells nor their function. These cell-specific effects require further investigation.

### Effects of senolytic treatment on formation of immunological memory and recall

**FIGURE 3:**
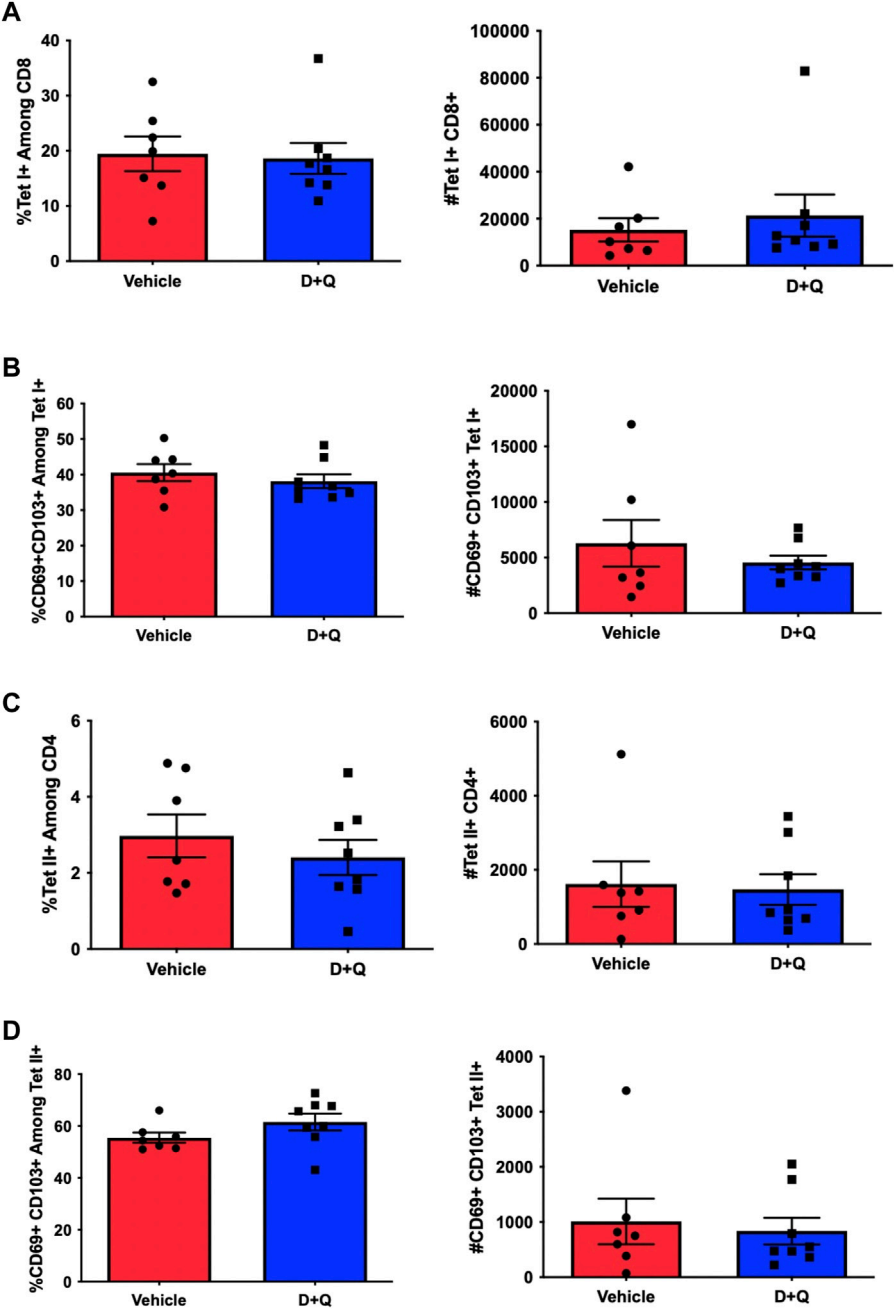
D + Q treatment does not affect generation of T-Cell memory. 18–20 months male mice were treated with D + Q or vehicle and subsequently infected with PR8 H1N1 flu. At 30 days post infection, lungs were assayed for frequency and number of flu NP-specific CD8 T-cells **(A)**. CD8 Tissue Resident memory cells (Trms), defined by CD69 and CD103 co-expression, was quantified **(B)**. Levels of flu NP-specific CD4 T-cells and CD4 Trms were also quantified **(C,D)**. Data are presented as mean ± standard error of the mean (SEM) and each symbol represents a single animal. Student’s *t*-test was utilized for all experiments, with results being considered significant at *p* < 0.05. Comparisons are nonsignificant unless otherwise indicated. *N* = 7–8 per group.

**FIGURE 4:**
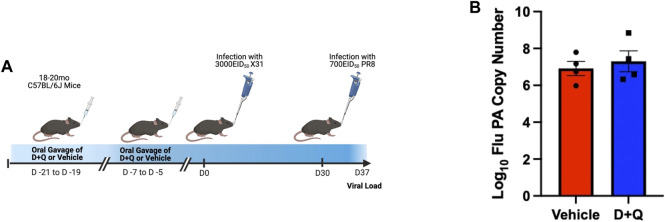
D + Q treatment does not improve recall response following secondary challenge. 18–20 months male mice were treated with D + Q or vehicle and subsequently infected with x31 H3N2 flu. At 30 days post infection, mice were rechallenged with PR8 H1N1 flu **(A)**. 7 days following rechallenge, viral load was quantified in the lungs via RT-qPCR for the flu acid polymerase (PA) gene **(B)**. Data are presented as mean ± standard error of the mean (SEM) and each symbol represents a single animal. Student’s *t*-test was utilized with results being considered significant at *p* < 0.05. Comparisons are nonsignificant unless otherwise indicated. *N* = 4 per group.

Prior efforts to assess the efficacy of senolytics to improve immune responses, by our group and others, have focused on primary time points following infection (Camell et al., 2021; Lorenzo et al., 2022). However, it is important to fully assess the effects of senolytic treatment across time. A key hallmark of the adaptive immune system is the ability to form immunological memory which is poised to respond more robustly upon pathogen re-encounter. To determine the ability of senolytic treatment to alter the differentiation of memory cells, we assessed the presence of flu-specific CD8 T-cells in the lungs at 30 DPI. Complete gating strategy can be found in Supplementary Figure S1. There were no significant differences found in frequency or number of NP-specific CD8s at this time point (Figure 3A). There was also no observed difference in the development of CD69^+^ CD103^+^ Tissue Resident memory (Trm) CD8 T-cells (Figure 3B). Similarly, no differences were observed in the CD4 T cells in the overall NP-specific or the Trm compartments (Figures 3C, D, respectively). In order to probe the potential function of these cells, we assayed for expression of PD-1, a marker of T-cell exhaustion (Supplementary Figure S3). D + Q treatment did not affect PD-1 expression and thus, did not improve the effects of aging on T-cell exhaustion. We went on to examine the capacity of the immune system to respond to a second heterologous influenza virus challenge (Figure 4A). There was no observed difference in the ability of D + Q treated animals to clear virus compared to control at 7 days post rechallenge (Figure 4B). Taken together, these results indicate the use of D + Q as a senolytic does not affect the overall immune response to flu infection.

## Discussion

This study demonstrates that use of D + Q as a senolytic fails to improve the overall response to influenza infection in aged mice. Considering our prior work using this model (Lorenzo et al., 2022), wherein we describe alleviation of age-related dysfunction in CD4 T-cell subset balance, this may indicate that D + Q induces both positive and negative effects that together do not impact the most general aspects of the immune response to infection such as pathogen load and antibody production. While not reaching significance, D + Q treatment induced a trending deficit in CD8 T-cell infiltration during a crucial time point. Perhaps this trending reduction may mitigate any potential benefits of the improved CD4 T-cell subset balance. It is possible that improved CD4 T-cell subset balance is not able to overcome the deficits in aged immune system in general. While our results do not suggest D + Q to be efficacious in potentiating aged immune responses, there still is great promise to further investigation of this therapeutic strategy.

Another similar study utilized senolytics to improve immune responses to an infection with murine hepatitis virus (Camell et al., 2021). This demonstrated that use of fisetin in aged mice conferred greater protection and increased antibody production following infection as a component of a model of polymicrobial exposure. This may indicate greater heterogeneity between specific senolytics in achieving improved immune outcomes. Further work may reveal dramatic differences between senolytics and in difference viral infections. Importantly, we did not observe improved serum antibodies with D + Q treatment at any time point, suggesting fisetin may have a more favorable effect on humoral responses compared to D + Q. Although differences between antibody quantification methods may also help partially explain this discrepancy. It is also possible that improvement in mortality following a primary infection may not correlate with increased efficacy of immunological memory. This remains to be investigated in the context of senolytic treatment with fisetin.

Therefore, there remains many open questions regarding the mechanisms by which clearance of senescent cells bring about changes in the immune system how those changes affect specific aspects of the immune response. These questions become more difficult when considering the full spectrum of immunity across both primary and memory responses. The immune system is very tightly regulated to potentiate pathogen clearance while fostering effective development of immune memory. It is possible that solely promoting primary effector responses will have unintended consequences in the memory response. It is imperative that future studies consider the effects of candidate drugs both at initial antigen encounter and upon a secondary challenge. Clinical trials are currently underway utilizing senolytic strategies to improve immune responses in older adults (NCT04476953, NCT04771611). Results from these trials may help elucidate the complex interactions between senescent cells and the immune system and how to best improve responses in this vulnerable population. One cannot dispute the great promise of senolytics and their potential benefits, but there remains much to be understood regarding the nuances between various senolytic strategies and their efficacy in various disease states or during different infections.

## Data Availability

The original contributions presented in the study are included in the article/Supplemental Materials, further inquiries can be directed to the corresponding author.

## Supplementary material

SUPPLEMENTARY FIGURE S1 Pro-inflammatory cytokine quantification 18–20 months male mice were treated as in Figure 1. At indicated time points, bronchoalveolar lavage (BAL) was collected and levels of IL-6, TNF-α, and CCL2 (**A-C**, respectively) were quantified via multiplex ELISA. N = 4–5 per group.

SUPPLEMENTARY FIGURE S2 Flow cytometric gating strategy for memory T-Cell analysis memory T-cell quantifications shown in Figure 3 were performed using the gating strategy shown.

SUPPLEMENTARY FIGURE S3 Exhaustion marker expression on CD4 and CD8 memory T-cells 18–20 months male mice were treated as in Figure 3. At 30 days post infection, lungs were assayed for expression of PD-1 by tissue resident memory CD8 and CD4 T-cells (**A,B** respectively) via flow cytometry. N = 7–8 per group.
