# Supplementary figures and images for: Senolytic treatment with dasatinib and quercetin does not improve overall influenza responses in aged mice
Source: Front Aging. 2023 Jun 16;4:1212750. doi: 10.3389/fragi.2023.1212750 (PMC10313122; doi:10.3389/fragi.2023.1212750)

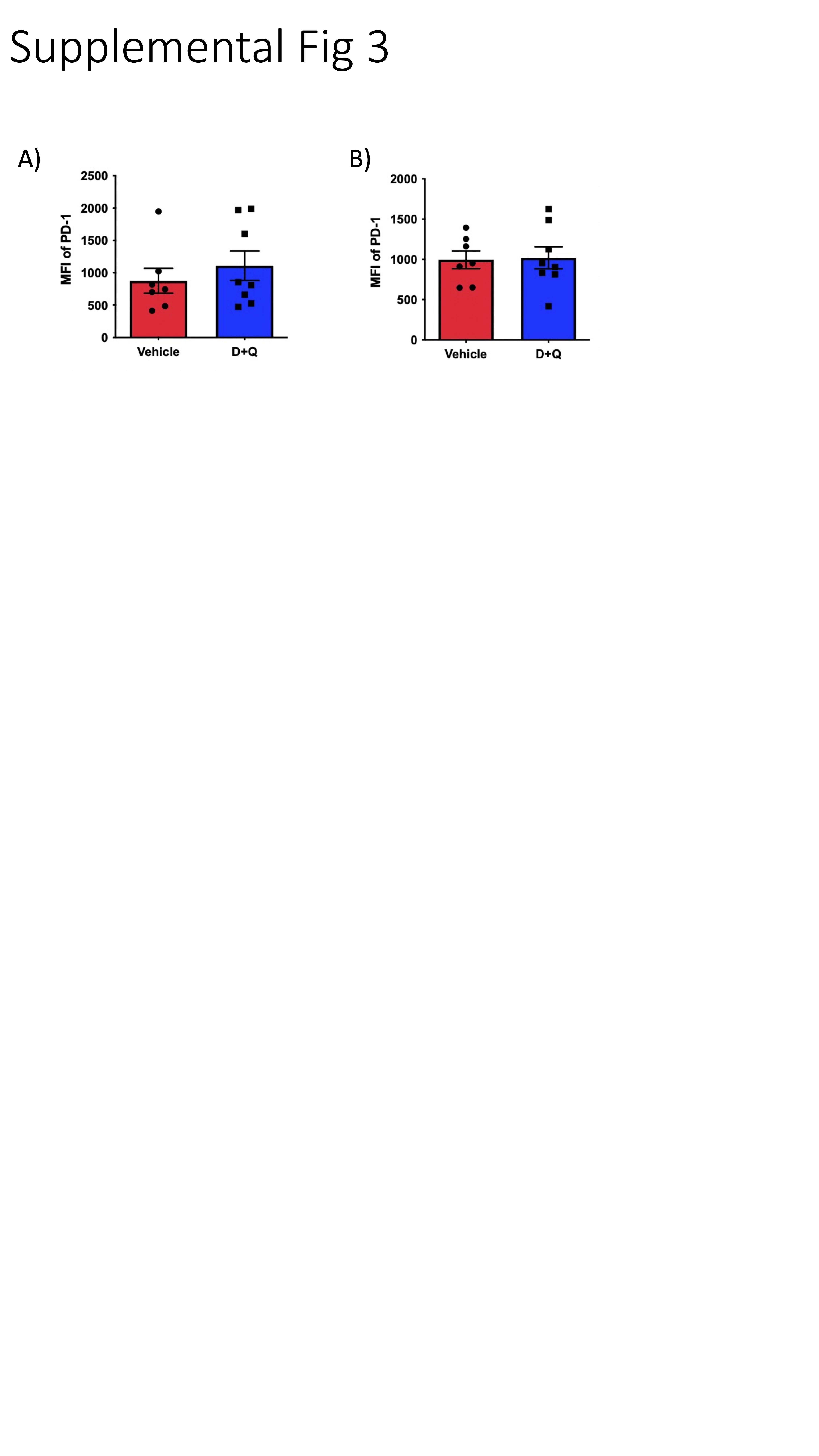

Supplement: Supplementary file 1 [file Image3.jpg]

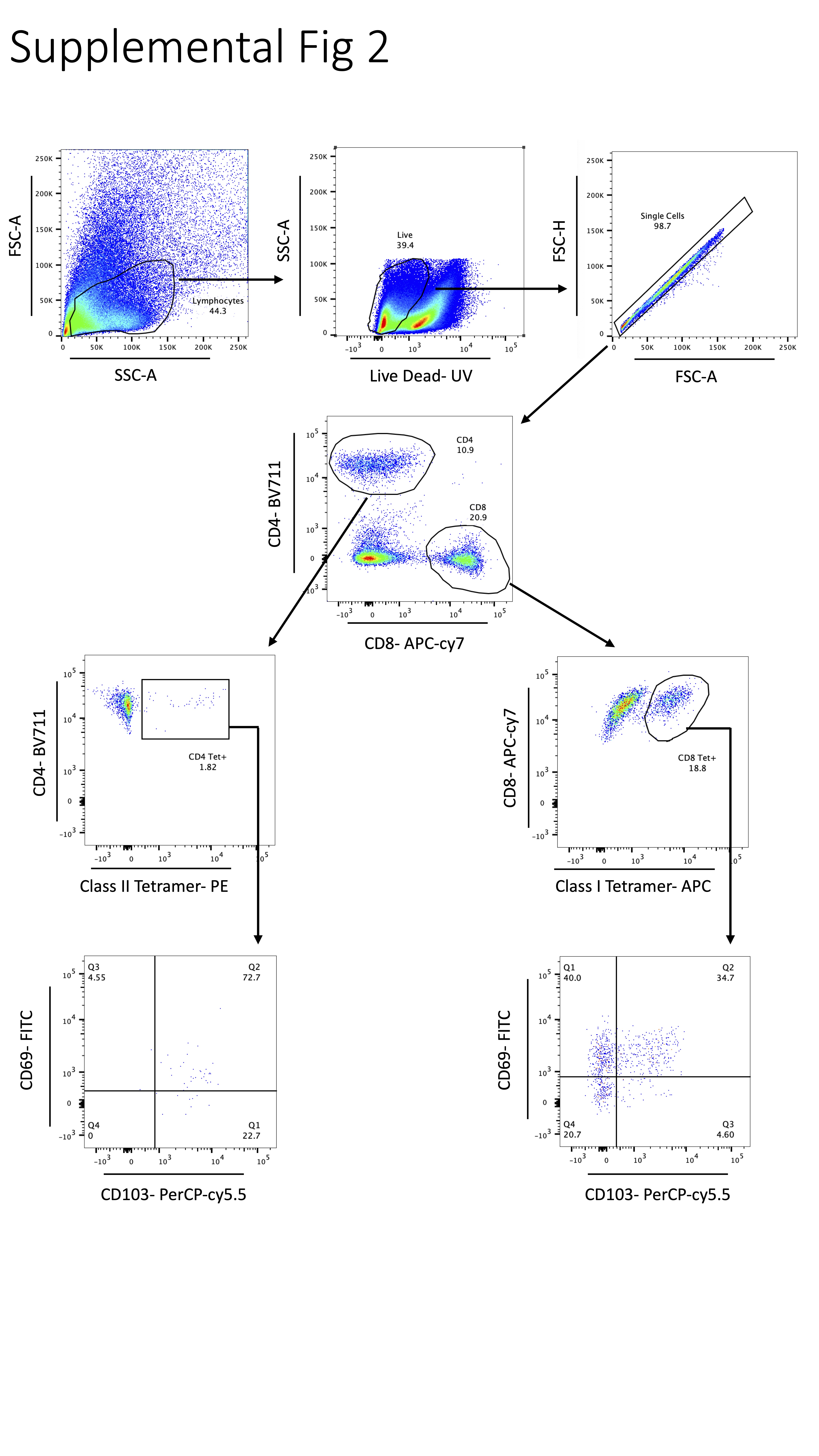

Supplement: Supplementary file 2 [file Image2.jpg]

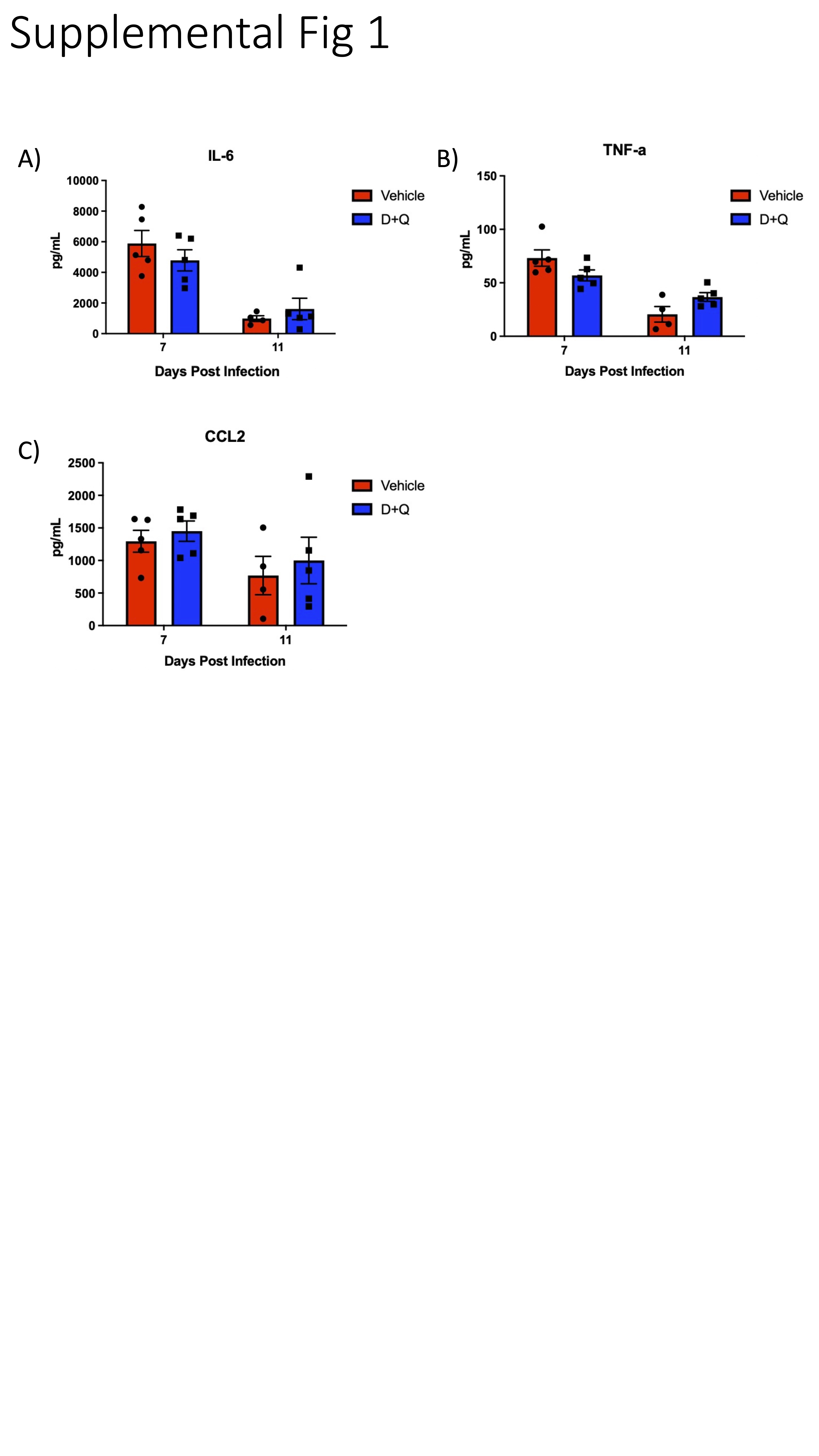

Supplement: Supplementary file 3 [file Image1.jpg]
